# High-dose IL-2 in metastatic melanoma: better survival in patients who also received patient-specific autologous tumor cell vaccine

**DOI:** 10.1186/2051-1426-1-S1-P208

**Published:** 2013-11-07

**Authors:** Robert O Dillman, Carol DePriest, Stephanie E McClure

**Affiliations:** 1 Hoag Institute for Research and Education, Hoag Hospital, Newport Beach, CA, USA; 2 Hoag Family Cancer Institute, Hoag Hospital, Newport Beach, CA, USA; 3 Cancer Biotherapy Research Group, Newport Beach, CA, USA

## 

Treatment with high-dose Interleukin-2 (IL-2) has been associated with long-term survival in small proportion of metastatic melanoma patients. We recently reported a median survival of 15.6 months, and a 20% 5-year survival rate for 150 such patients who were hospitalized for high-dose i.v. IL-2 between May 1987 and April 2010. [1] A recent report showed a survival advantage for the addition of gp100 vaccine plus high-dose IL-2 compared to treatment with IL-2 alone [2]. We were aware that several of our IL-2 patients had also received patient-specific tumor cell vaccines derived from autologous tumor cell lines. We wished to determine whether this may have contributed to their high 5-year survival rate. Comparison of existing data bases revealed that 27 of the 150 IL-2 patients had also received a patient-specific vaccine; 123 had not. The table below (Table 1) summarizes survival data, which was calculated from the date IL-2 was initiated. Survival was much better in patients who received a patient-specific vaccine in addition to IL-2 (p<0.001). That group was also younger, but age was not a predictor of survival in as much as median and 5-year survival rates were not dissimilar: 14.1 months and 23% for patients age <50 years at the time of IL-2, compared to 15.9 months and 17% for patients > age of 50 (NSD). Of the 27 vaccine patients, 7 started vaccine therapy an average of 8.7 mos. before receiving IL-2 (range 2.4 to 40 mos.) and 20 received vaccine a median of 14.2 months after starting IL-2 (range 1 to 42 mos.); 12 received injections of irradiated autologous tumor cells and 15 received injections of dendritic cells loaded with antigens from irradiated autologous tumor cells, and suspended in 500 microgram GM-CSF. Survival was longer in patients who received IL-2 first (5-yr survival 55% vs 14%), and in patients who received the dendritic cell vaccine (5-yr survival 53% vs 33%). This analysis suggests that receipt of high-dose IL-2 followed by a patient specific vaccine results in better survival than IL-2 alone, but the limitations of such a retrospective analysis, and the risk of confounding unintended bias, are significant.

**Table 1:** 

Therapy	IL-2	IL-2 + Vaccine
Portion female	58%	59%
Median age at diagnosis	50 yrs	40 yrs
Median age at IL-2	54 yrs	44 yrs
Median age at vaccine	N/A	47
Median survival from IL-2	12.8 mos	40.3 mos
5-year survival from IL-2	14%	44%
